# Prediction of depression in European general practice attendees: the PREDICT study

**DOI:** 10.1186/1471-2458-6-6

**Published:** 2006-01-12

**Authors:** Michael King, Scott Weich, Francisco Torres-González, Igor Švab, Heidi-Ingrid Maaroos, Jan Neeleman, Miguel Xavier, Richard Morris, Carl Walker, Juan A Bellón-Saameño, Berta Moreno-Küstner, Danica Rotar, Janez Rifel, Anu Aluoja, Ruth Kalda, Mirjam I Geerlings, Idalmiro Carraça, Manuel Caldas de Almeida, Benjamin Vicente, Sandra Saldivia, Pedro Rioseco, Irwin Nazareth

**Affiliations:** 1 Department of Mental Health Sciences, UCL, London, UK; 2 Division of Health in the Community, University of Warwick, Coventry, UK; 3 Department of Psychiatry, University of Granada, Granada, Spain; 4 Dept. of Family Medicine, University of Ljubljana, Ljubljana, Slovenia; 5 Faculty of Medicine, University of Tartu, Tartu, Estonia5; 6 University Medical Center, Utrecht, Netherlands; 7 Faculdade Ciências Médicas, University of Lisbon, Lisbon, Portugal; 8 Department of Primary Care and Population Sciences, UCL, London, UK; 9 Department of Family and Community Medicine, Malaga, Spain; 10 Encarnação Health Centre, Lisbon, Portugal; 11 Mora Health Centre, Mora, Portugal; 12 Departamento de Psiquiatría y Salud Mental, Universidad de Concepción, Concepción, Chile; 13 Department of Primary Care and Population Sciences, UCL and Scientific Director, Medical Research Council General Practice Research Framework, UCL, London, UK

## Abstract

**Background:**

Prevention of depression must address multiple risk factors. Estimating overall risk across a range of putative risk factors is fundamental to prevention of depression. However, we lack reliable and valid methods of risk estimation. This protocol paper introduces PREDICT, an international research study to address this risk estimation.

**Methods/design:**

This is a prospective study in which consecutive general practice attendees in six European countries are recruited and followed up after six and 12 months. Prevalence of depression is assessed at baseline and each follow-up point. Consecutive attendees between April 2003 and September 2004 who were aged 18 to 75 were asked to take part. The possibility of a depressive episode was assessed using the Depression Section of the Composite International Diagnostic Interview. A selection of presumed risk factors was based on our previous work and a systematic review of the literature. It was necessary to evaluate the test-retest reliability of a number of risk factor questions that were developed specifically, or adapted, for the PREDICT study. In a separate reliability study conducted between January and November 2003, consecutive general practice attendees in the six participating European countries completed the risk factor items on two occasions, two weeks apart. The overall response rate at entry to the study was 69%. We exceeded our expected recruitment rate, achieving a total of 10,048 people in all. Reliability coefficients were generally good to excellent.

**Discussion:**

Response rate to follow-up in all countries was uniformly high, which suggests that prediction will be based on almost a full cohort. The results of our reliability analysis are encouraging and suggest that data collected during the course of PREDICT will have a satisfactory level of stability. The development of a multi-factor risk score for depression will lay the foundation for future research on risk reduction in primary care. Our data will also provide the necessary evidence base on which to develop and evaluate interventions to reduce the prevalence of depression.

## Background

Depression will rank second to cardiovascular disease as a global cause of disability by 2020 [1]. It occurs in up to a quarter of general practice attendees [2,3], relapse is frequent up to 10 years from first presentation [4-6] and residual disability is common [7]. Prevalence is determined by exposure to risk factors that precipitate or maintain episodes of disorder. The two most consistently identified risk factors are low socio-economic status [8-10] and female sex [11]. Relative poverty and unemployment are associated with longer duration of episodes of depression rather than their onset [10,12]. Socio-economic risk factors that might conceivably be addressed include low income and financial strain [10,13], unemployment [10], work stress [14], social isolation [14,15], and poor housing [9]. Fixed factors such as a family history of depression [12] and personality play a part [16] but it is not yet known whether they act independently of other risk factors.

Prevention of depression must address multiple risk factors [17], include those at low and moderate risk [18] and be acceptable to the target population [19]. However, in contrast to physical disorders such as cardiovascular disease, many mutable risk factors affect the duration of episodes of depression, rather than simply their onset [20].  Estimating overall risk across a range of putative risk factors is fundamental to prevention of depression. However, we lack reliable and valid methods of risk estimation [21].

The PREDICT study is taking place in six European and one Latin American country in order to test the following hypotheses: 1) A reliable and valid multi-factor scale can be developed to determine the risk for the onset and maintenance of depression in primary care attendees; and 2) The overall risk equation derived from data for all countries combined will have similar accuracy in predicting episodes of depression for each country. In this introductory paper, we describe the method, response rates at baseline and first follow-up and the reliability of instruments developed or adapted for the study.

## Methods/design

### Design

This is a prospective study in which consecutive general practice attendees are recruited and followed up after six and 12 months. Prevalence of depression is assessed at baseline and each follow-up point. The study was approved by the relevant ethical committees in each country.

### Setting

Six European and one Latin American centre are participating: 1) 25 general practices in the Medical Research Council's General Practice Research Framework, distributed across the United Kingdom; 2) nine large primary care centres in Andalucía, Southern Spain; 3) 74 general practices distributed nationwide in Slovenia; 4) 23 general practices distributed nationwide in Estonia; 5) seven large general practice centres near Utrecht, The Netherlands; 6) two large primary care centres in urban and rural areas of Portugal that include 25 general practitioners; and 7) 78 general practices in Chile. The general practices taking part extend over urban and rural settings in each country and populations with considerable socio-economic and ethnic variation.

### Sample

Consecutive attendees between April 2003 and September 2004 who were aged 18 to 75 were asked to take part. Those over 75 were excluded because prevalence of cognitive impairment increases after that age. Other exclusion criteria were an inability to understand one of the principal languages involved, severe organic mental illness and terminal illness. Participants who gave informed consent subsequently undertook an interview at their home or the general practice within two weeks. Because of local service preferences the recruitment approach was slightly different in each country. In the UK and the Netherlands, researchers approached patients waiting to see the doctor, while in the other countries the doctors raised the idea of the research first before the researcher was introduced.

### Measures of outcome and exposure

#### Depression

The possibility of a depressive episode was assessed using the Depression Section of the Composite International Diagnostic Interview (CIDI) [22,23], which provides six month and lifetime psychiatric diagnoses according to ICD10 and DSMIV.

#### Risk factors for the depression

Selection of presumed risk factors was based on our previous work [24,25] and a systematic review of the literature. Where possible, we used published self-report measures of established reliability and validity. In some instances, questions were developed for the study or adapted from available standardised instruments. We addressed risk factors that are intrinsic either to the individual or to the social context, while remaining aware that there is inevitable overlap in such a categorisation. The risk factors in italics were assessed for test-retest reliability (see below).

• Socio-demographic factors.

• A lifetime history of depression (assessed by CIDI at baseline).

• *Controls, demands and rewards for unpaid work using an adapted version of the job content instrument *[26].

• Debt and financial strain [10].

• Consultation rate in the general practice [27].

• Self-rated physical health problems and limiting long-term disability using the Short Form 12, a brief, self-report disability schedule that has application across a number of cultures [28]

• Alcohol misuse using the WHO's AUDIT questionnaire [29]

• *Use of recreational drugs adapted from the relevant sections of the CIDI*.

• Brief questions on cigarette consumption

• For women, questions on menstruation, pregnancy and childbirth from the Patient Health Questionnaire (PHQ) [30].

• *Brief questions on the quality of sexual and emotional relationships adapted from a standardized questionnaire *[31].

• *Problems in people close to participants *[32].

• Childhood experiences of physical, emotional and sexual abuse [33]

• Nature and strength of spiritual beliefs [34].

• *Family psychiatric history: depression in first-degree family members requiring pharmacological or psychological treatment in primary or secondary care. Suicide in first degree relatives *[35].

• Anxiety symptoms using the anxiety section of the PHQ [30].

• One question on whether or not, and at what age, the participant had lost one or both parents by death.

• Household type and composition.

• *The living environment including satisfaction with neighbourhood and perception of safety inside/outside of the home, using questions from the Health Surveys for England *[36].

• Threatening life events in the preceding six months, using a brief validated checklist [37].

• *Experiences of discrimination based on a recent European study *[38].

• Adequacy, availability and sources of social support [39].

### Assessment of test-retest reliability

Many of the items in the PREDICT risk factor assessment are either based on previously validated measures, or concern exposures that are likely to be reported with a high degree of reliability (e.g. age, sex, ethnicity and civil status). However, we needed to evaluate the test-retest reliability of a number of risk factor questions (noted in italics above) that were developed specifically, or adapted, for the PREDICT study. In a separate reliability study conducted between January and November 2003, consecutive general practice attendees in the six participating European countries were invited to complete the risk factor items on two occasions, two weeks apart. At the time of retest, we re-contacted participants (using the general practice/health centre letterheads), reminding them of the study. Questionnaires were completed by assisted interviews. Expert opinions regarding the appropriate interval between test and retest vary from an hour to a year, depending on the task; a test-retest interval of between two and 14 days is usual [40]. Two weeks is sufficient time for patients to have forgotten their first responses but for opinions to have remained stable. We did not attempt to estimate validity of these measures, given that 1) there are many uncertainties in choosing a standard against which to validate patient reports of this type, and 2) patients' reports will form the basis of the eventual risk tool.

### Data quality control

Data quality was monitored to ensure that the project yielded data of the highest validity and reliability.

#### Translation of instruments

We used standardised validated instruments available in the native language of all the participant countries. In those instances where this was not possible we translated standardised instruments from English to the relevant languages. Where we developed our own measures, these were also translated from English into the languages of the participant countries. Each translation was back-translated by professional translators and the penultimate version verified by the co-ordinating centre. No major discrepancy was identified in any of the back-translations.

#### Data checking

Locally, each interview was checked for completion by the interviewer. Quality assurance focused on the standardised training of researchers in the use of the CIDI and other questionnaires, in the recruitment and interviewing of patients and in data management. Over and above national team meetings a research coordinator made two assessments of each interviewer during the baseline interviews to monitor the interview process, assess adherence to the CIDI, provide structured feedback for improvement and manage other problems as they arose. Structured and standardised data quality control sheets are used to manage data and ensure its transfer to the coordinating centre (UK). Progress reports for each national centre are submitted every six months and critically assessed by the steering group at project management meetings. Each participating country double entered 10% of its data records and accepted a 1% error rate before deciding on full double entry.

### Statistical analysis

We calculated test-retest agreement using the kappa statistic for questions with two response options and the intraclass correlation coefficient (ICC) for items with more than two ordinal categories. When both follow-ups are complete we shall 1) be able to identify risk factors for incidence of depression over six and 12 months, from participants who were not depressed at baseline; 2) be able to identify factors for recovery from depression over six and 12 months, from participants who were depressed at baseline; have extra data with which to predict episodes of depression over 6 months, by relating not only data available from baseline and 6 month time points, but also from 6 month to 12 month time points; 3) be able to determine time of onset and offset of episodes with greater precision and reliability over intervals of six, rather than 12 months; and 4) determine how incidence of, and recovery from, depression is associated with changes in risk factors over 6 and 12 months. We shall derive risk factor equations using logistic regression analysis on a randomly chosen 50% sample (training set). We shall then apply the equation for risk to the remaining 50% (test set). Actual occurrence of depression during follow up will be compared with the prediction using relative operating characteristics (ROC) curve analysis. We shall choose the point of the ROC curve corresponding to 70% specificity as a cut-off for estimating sensitivity for subjects in all countries combined (and for participants in each country) in the test set. Confidence intervals for sensitivity in each country will indicate country heterogeneity. If estimated sensitivity in a particular country is significantly worse than overall sensitivity, and this difference is clinically important, new risk factor equations will be derived which include country specific effects. The latter can include an allowance for differences in overall case rate, or varying impacts of certain risk factors. We shall test the new equation until no further reduction in heterogeneity is possible. If after developing the best possible equation, sensitivity is still substantially worse than 70% for any country at the 1% significance level, a new equation will be derived specifically for that country. We believe this is unlikely to prove necessary, as there is no reason to suspect that the model will differ across countries, given our wealth of knowledge about risk factors.

### Statistical power and sample size

At the time our sample size was calculated, Chile's participation was not finalised and thus it was estimated on the basis of six participating countries. A DSMIV diagnosis of major depression will provide the primary outcome measure. Our estimate of numbers for the prospective study was based on 1) a specificity and sensitivity of our risk score of at least 70%; 2) an assumption of a case rate of depression of approximately 15% and no major heterogeneity between centres. This requires a sample size of 2193, which we then doubled to allow for development of the risk factor score on one random half of the population and testing on the other, and to allow for an attrition rate of 30%. Thus our target recruitment was 6266 or 1044 in each country. In evaluating test-retest reliability, we calculated item coefficients for all European countries combined. We aimed to recruit at least 200 participants to achieve an intraclass correlation coefficient (ICC) with 95% confidence intervals of ± 0.10, provided the true reliability exceeds 0.58 [40].

## Results

### Response rates

The overall response rate at entry to the study was 69%, with the lowest rates in the UK and the Netherlands and the highest in Chile (table 1). We exceeded our expected recruitment rate, achieving a total of 10,048 people in all. Response rates at the six month follow-up point were very high; 12 months follow-up is not yet complete.

### Data quality

The baseline error rates for data entry in each country were well below the 1% level of acceptability (table 2).

### Test-retest reliability assessment of risk factor questions

285 general practice attendees (152 women and 133 men) completed the questions on two occasions. Numbers in each country ranged from 40 in Slovenia to a maximum of 67 in the Netherlands. Their mean age was 44.6 years (SD 16.0), which was close to the mean age of the eventual study population. Reliability coefficients were generally good to excellent [41,42] (table 3). Questions on unpaid work generated kappa and ICC in the fair to good range (0.59 to 0.70), except for one question concerning how often participants get help and support with unpaid work difficulties. This question also had relatively poor percentage agreement. Five of the six questions on recent discrimination had kappa coefficients in the fair to excellent range. Responses to the sixth, concerning discrimination on the grounds of skin colour, were skewed due to the small number of non-white participants. As a consequence the kappa coefficient was low, but there was very high percentage agreement.

## Discussion

Most research into depression in primary care populations has focused on management of current disorders rather than prediction of risk or prevention of future episodes. When our follow-up is complete we shall be able to report on whether a risk assessment is possible in a general practice setting. Our main results to date are 1) that response rates to follow-up at 6 months are high and 2) our instruments have acceptable reliability.

### Setting and response rates

Our study is based on general practice attendees and not on a probabilistic sample recruited in the community. However, most people with depression visit their GP, although many will not complain of depression and nor will their mood disorder be recognized [2]. Thus the epidemiology of depression in general practice closely mirrors that seen in the community, with the caveat that prevalence rates are higher in the former [2]. Although response rates at baseline were lower in the UK and The Netherlands than in other countries, response rate to follow-up in all countries is uniformly high, which suggests that prediction will be based on almost a full cohort. The lower response rate in the UK and the Netherlands may reflect the different recruitment process we undertook in those countries, in which the study was not so obviously endorsed by the GP. There may also be differences in the public's attitudes to research in those two countries, where recruitment is generally lower across a range of research. There were also differences in the geographical distribution of participating general practices in each country, some being more nationally extended than in others. This difference reflected the varying opportunities and networks available to the centres. We shall take account of this variation, particularly urban-rural differences, in our analysis of risk.

### Data quality and reliability

Our data monitoring and management has ensured that data quality reaches a high standard across the centres. The results of our reliability analysis are encouraging and suggest that data collected during the course of PREDICT will have a satisfactory level of stability. Reliability increases with sample size and thus we also know that estimates reported here are more conservative than will be the case in the main study. The two week period used in this test-retest evaluation may not be equally appropriate for all questions. For example, answers to questions on unpaid work will have less stability over this time than those to questions on family history of psychological disorder or the living environment, since satisfaction and control at work depend on challenges and interactions that may change daily. A question on how often participants get help and support with unpaid work difficulties exhibited only moderate stability between test and retest and relatively poor percentage agreement. Thus, we shall not it include in the final analysis of prospective data. The question on racial discrimination will be retained as its reliability could not be assessed fairly in this data set.

### Significance of the study

Depression accounts for one-fifth of all consultations with GPs [43]. Those affected experience similar levels of excess mortality [44] and reduced quality of life as people with chronic physical disorders [45]. The aim of our study is to break new ground by quantifying the future risk of episodes of depression in primary care settings. The development of a multi-factor risk score for depression will lay the foundation for future research on risk reduction in primary care. Just as in prevention of cardiovascular disease [46], effective interventions for depression will need to address multiple risk factor domains, extend to those at low or moderate risk and be acceptable to the target population. Our data will also provide the necessary evidence base on which to develop and evaluate interventions to reduce the prevalence of depression. In so doing up to 15% of people attending general practitioners will potentially benefit by identification of their risk for episodes of depression, with the consequent reduction of distress and absence from work.

## Competing interests

The author(s) declare that they have no competing interests.

## Authors' contributions

MK and IN originated the idea for the study, led on its design, obtained funding and coordinated the project and analysis of data. MK led on writing the paper and is the guarantor for the study. SW participated in the design of the study, and read and approved the final manuscript. RM participated in the design of the study and analysis of data for the manuscript. He also read and approved the final manuscript. CW participated in the recruitment of patients and the overall coordination of the project and helped to collect and analyse data for the paper. He also participated in writing the paper.

FT participated in the design of the study design and coordination of the research at the participating centre. JAB and BM were involved in discussing study design, contributing to Spanish data collection and commenting on the results. All contributed to the paper.

IŠ participated in the design of the study and supervised the study in Slovenia. DRP coordinated the study in Slovenia. JR performed data checking for the Slovenian sample.

HIM participated in the project design and coordination. AA participated in the study design and managed the Estonian data collection. RK participated in the enrolment of GPs and patients and helped to perform Estonian data collection. All read and approved the final manuscript.

JN participated in the study design and coordination of the research in the Dutch context. MIG supervised and participated in the Netherlands data collection and data management and was involved in revising the manuscript.

MX participated in the design of the study, coordinated data collection in Portugal. IC and MCA collected and managed the Portuguese data. All three authors read and approved the manuscript.

BV supervised different stages of the study and contributed to the paper. SS coordinated the field study, performed data management and contributed to the paper. PR assisted in collection and management of data. All authors read and approved the final manuscript.

## Pre-publication history

The pre-publication history for this paper can be accessed here:



## Figures and Tables

**Table 1:** Response rates

**Country**	**Numbers approached**	**Numbers refused**	**Numbers not** **eligible**	**Total interviewed** ** at baseline** **(% of eligible)**	**Total interviewed** **6 months** **(%of baseline)**
**UK**	3319	1681	313	1325 (44%)	1144 (86%)
**Spain**	1470	194	6	1270 (87%)	1008 (79%)
**Slovenia**	1405	276	10	1119 (80%)	1036 (93%)
**Estonia**	1370	270	6	1094 (80%)	1025 (94%)
**The Netherlands**	3089	1478	390	1221 (45%)	1162 (95%)
**Portugal**	1552	369	3	1180 (76%)	1049 (89%)
**Chile**	3000	82	79	2839 (97%)	2580 (91%)
**All countries**	**15205**	**4444**	**713**	**10048 (69%)**	**9004 (90%)**

**Table 2:** Data entry error rates

**Country**	**Total no.** **of Questionnaires** **checked (A)**	**Total min no.** **of items per** **participant (B)**	**Total min no.** **of items input** **(A × B)**	**1% level of** **input errors (A × B)** **× 0.01**	**No of input** **errors made**
**UK**	131	187	24497	245	39
**Spain**	127	187	23749	238	32
**Slovenia**	112	187	20944	209	116
**Estonia**	110	187	20570	206	63
**Netherlands**	121	187	22627	226	43
**Portugal**	125	187	23375	234	22
**Chile**	2839	187	530,893	5309	2495

**Table 3:** Reliability analysis by questionnaire

Questionnaire	Analyses	Reliability range of items not removed		Percent agreement range	
		Min	Max	Min	Max
Family History of Psychological Disorder	Kappa and ICC	0.70	1.00	82.35	100
Recreational Drug Use	Kappa	0.64	0.98	88.21	100
Unpaid Work	Kappa and ICC	0.59	0.70	67.14	86.94
Living Environment	ICC	0.59	0.74	72.34	75.80
Discrimination	Kappa	-0.01	1.00	96.38	100
Relationships with Others	ICC	0.64	0.73	64.84	83.67
Difficulties with Persons Close to you	Kappa	0.65	0.84	93.42	96.94
